# Architectural Tuning of Redox-Responsive Copolymer Hydrogels: Fast Gelation, Self-Healing, and Superior Mechanics via Block vs Random Networks

**DOI:** 10.1021/acspolymersau.5c00166

**Published:** 2026-02-10

**Authors:** Dhayanithi Senthilkumar, Yun-Jie Liao, Shr-Shiang Weng, Chih-Yu Kuo

**Affiliations:** † Department of Chemical Engineering and Biotechnology, 34877 National Taipei University of Technology, Taipei 10608, Taiwan; ‡ International Graduate Program of Energy and Optoelectronic Materials Program (EOMP), National Taipei University of Technology, Taipei City 10608, Taiwan; § Institute of Biochemical and Biomedical Engineering, National Taipei University of Technology, Taipei City 10608, Taiwan; ∥ High-Value Biomaterials Research and Commercialization Center, National Taipei University of Technology (Taipei Tech), Taipei 10608, Taiwan

**Keywords:** RAFT polymerization, redox-responsive hydrogel, disulfide bond, block copolymer, random copolymer

## Abstract

Stimulus-responsive hydrogels have emerged as promising candidates for next-generation biomedical materials, yet the direct influence of copolymer architecture on their gelation kinetics, mechanical performance, and adaptive properties remains underexplored. Here, we systematically compare random (poly­(HEMA-*co*-NIPAM)) and block (poly­(HEMA-*b*-NIPAM)) copolymer architectures, synthesized via RAFT polymerization and cross-linked with disulfide-containing DTPA, to engineer redox-responsive hydrogels. Notably, random copolymer hydrogels achieve ultrafast gelation within 30 s and display superior elasticity, with a fracture strain of 295.9% at 40 wt % solid content-substantially higher than the 99.0% observed in block copolymer hydrogels. Thermal analysis reveals that random copolymer hydrogels exhibit a maximum degradation temperature of 380 °C, surpassing the 340 °C of block counterparts, while DSC shows a higher glass transition temperature (135 °C vs 125 °C). SEM imaging further demonstrates that random hydrogels possess uniform, interconnected pores (∼20–25 μm), whereas block architectures yield irregular and larger pores (∼35–40 μm). All hydrogels display robust self-healing and reversible gel–sol–gel transitions upon redox cycling, attributable to dynamic disulfide linkages. These results underscore the pivotal role of macromolecular architecture in tuning hydrogel performance, and establish random copolymer networks as promising platforms for smart wound dressings and responsive drug delivery.

## Graphical abstract

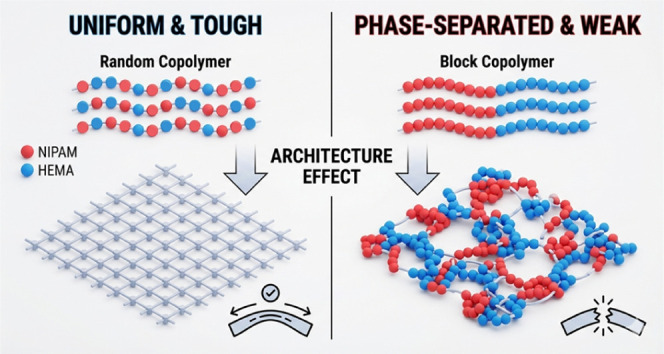

## 1 Introduction

Hydrogels, which possess three-dimensional polymeric networks with high water content, are central to many biomedical innovations, including wound dressings, drug delivery, and tissue engineering, because they are biocompatible and can closely mimic the extracellular matrix.^1^ In recent years, the development of stimuli-responsive hydrogels, capable of adapting their physical and chemical properties in response to environmental cues such as pH, temperature, light, or redox potential, has created new possibilities for designing smart biomedical materials that can provide on-demand, site-specific action in complex biological environments.^2^

The particular promise of redox-responsive hydrogels lies in their ability to use dynamic disulfide linkage, which allow for reversible transitions between gel and sol states when exposed to endogenous redox gradients present in diseased or healing tissues.^3^ This property enables hydrogels to achieve controlled drug release, self-healing,^4^ and adaptive mechanical behavior,^5^ all of which are directly matched to the biochemical microenvironment relevant for medical applications.^6^ Although the incorporation of redox-responsive chemical groups has been widely explored, the performance and dynamic behavior of these hydrogels are strongly dependent on the macromolecular architecture that organizes the functional monomers within the polymer backbone.^7^ What remains insufficiently understood is the precise structure–property relationship that connects polymer architecture with the efficiency and nature of redox responsiveness,^8^ gelation kinetics, network uniformity, and adaptive mechanical properties.^9^ Conventional research often focuses only on the presence of redox-labile bonds, but fails to recognize how the sequence and spatial distribution of these functional groups-whether arranged randomly or as well-defined blocks-affect the accessibility of cross-linking sites,^10^ the uniformity of the hydrogel network, and ultimately the macroscopic responsiveness and functional outcomes of the material.^11^

In response to this gap in knowledge, our study undertakes a comprehensive investigation into the ways in which copolymer architecture, specifically the contrast between block and random arrangements, directly influences the manifestation and efficiency of redox-responsiveness in hydrogels.^12^ By systematically synthesizing and comparing random copolymers of poly­(HEMA-*co*-NIPAM) and block copolymers of poly­(HEMA-*b*-NIPAM), each cross-linked with disulfide-containing DTPA, we clarify the links between molecular structure and key hydrogel properties. HEMA provides reactive –OH groups for covalent cross-linking with the disulfide-based DTPA, ensuring robust network formation.^13^ NIPAM serves as a hydrophilic monomer that could contribute thermoresponsive behavior via its lower critical solution temperature (LCST ∼32 °C).^14^ In the present work, all experiments were performed at room temperature (∼25 °C), below the LCST, to isolate the effects of cross-linking density and network architecture on gelation, mechanical properties, and redox responsiveness. These properties include gelation rate, mechanical strength, thermal stability, microstructural uniformity, and the capacity for reversible redox cycling and self-healing. The results of this work demonstrate that the interplay between macromolecular architecture and dynamic redox chemistry fundamentally determines the rational design of advanced, adaptive hydrogels (Scheme 1). The findings from this comprehensive structural comparison not only advance mechanistic understanding of redox-responsive materials, but also provide a framework for tailoring next-generation wound dressings and drug delivery systems in which both structural effects and stimuli-responsiveness are harmonized for optimal biomedical performance.^15^

**Scheme 1:**
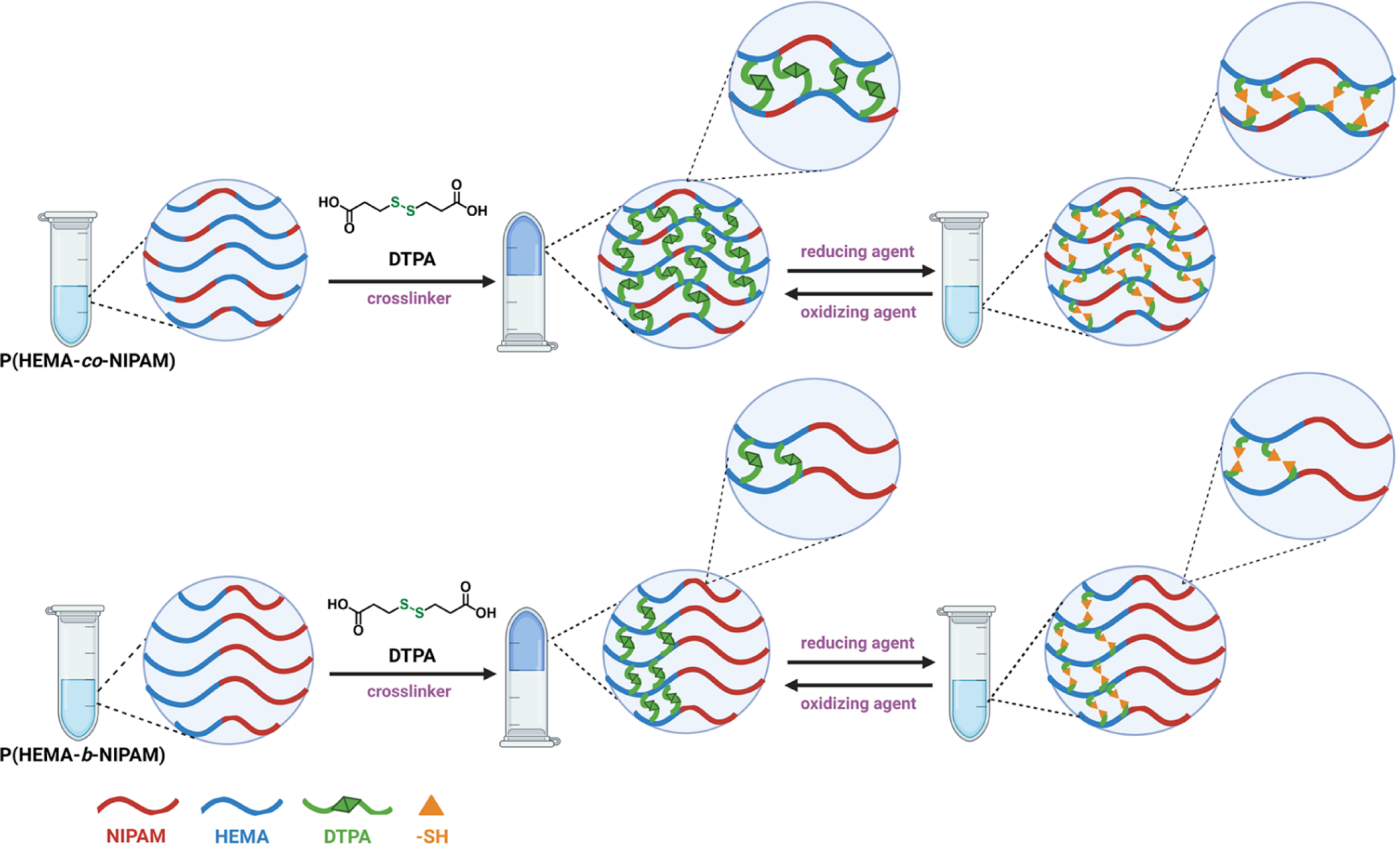
Schematic Representation of the Synthesis of Random and Block Copolymers via RAFT Polymerization Using HEMA and NIPAM Monomers, Followed by Disulfide Crosslinking Using DTPA to form Redox-Responsive Hydrogels

## 2 Experimental Procedures and Analytical Methods

### 2.1 Materials

2-hydroxyethyl methacrylate (HEMA), 2,2′-Azobis­(2-methylpropionitrile) (AIBN), and 3,3′-dithiodipropionic acid (DTPA) were purchased from TCI. *N*-Isopropylacrylamide (NIPAM), 1,4-dithiothreitol (DTT) were purchased from Thermo Scientific. 2-(Dodecylthiocarbonothioylthio)-2-methylpropionic acid (DDMAT), 1-(3-dimethylaminopropyl)-3-ethylcarbodiimide hydrochloride (EDC·HCl) were purchased from Merck. Organic solvents were obtained from Fisher Chemical. All chemical reagents are used directly as received without further purification.

### 2.2 Synthesis of Random Copolymer Poly­(HEMA-*co*-NIPAM)

*N*-Isopropylacrylamide (NIPAM, 4.0 g, 35.3 mmol), 2-hydroxyethyl methacrylate (HEMA, 0.5 g, 3.84 mmol), 2-(dodecylthiocarbonothioylthio)-2-methylpropionic acid (DDMAT, 32 mg, 0.088 mmol), and azobisisobutyronitrile (AIBN, 2.9 mg, 0.018 mmol) were introduced into a three-necked round-bottom flask and dissolved in 11.3 mL of anhydrous *N*,*N*-dimethylformamide (DMF). Prior to polymerization, the solution was thoroughly degassed under vacuum and purged with nitrogen for 30 min to eliminate dissolved oxygen. Polymerization was conducted at 70 °C for 16 h under continuous stirring at 300 rpm (Figure 1a). Upon completion, the reaction was quenched by cooling the flask in an ice bath (0 °C), and the crude polymer solution was subsequently dialyzed against methanol for 24 h and then deionized water for an additional 48 h to remove residual monomers and solvent.^16^ The purified product was collected by freeze-drying over 72 h to yield the amphiphilic random copolymer P­(HEMA_44_-*co*-NIPAM_402_), hereafter referred to as r402.

**Figure 1:**
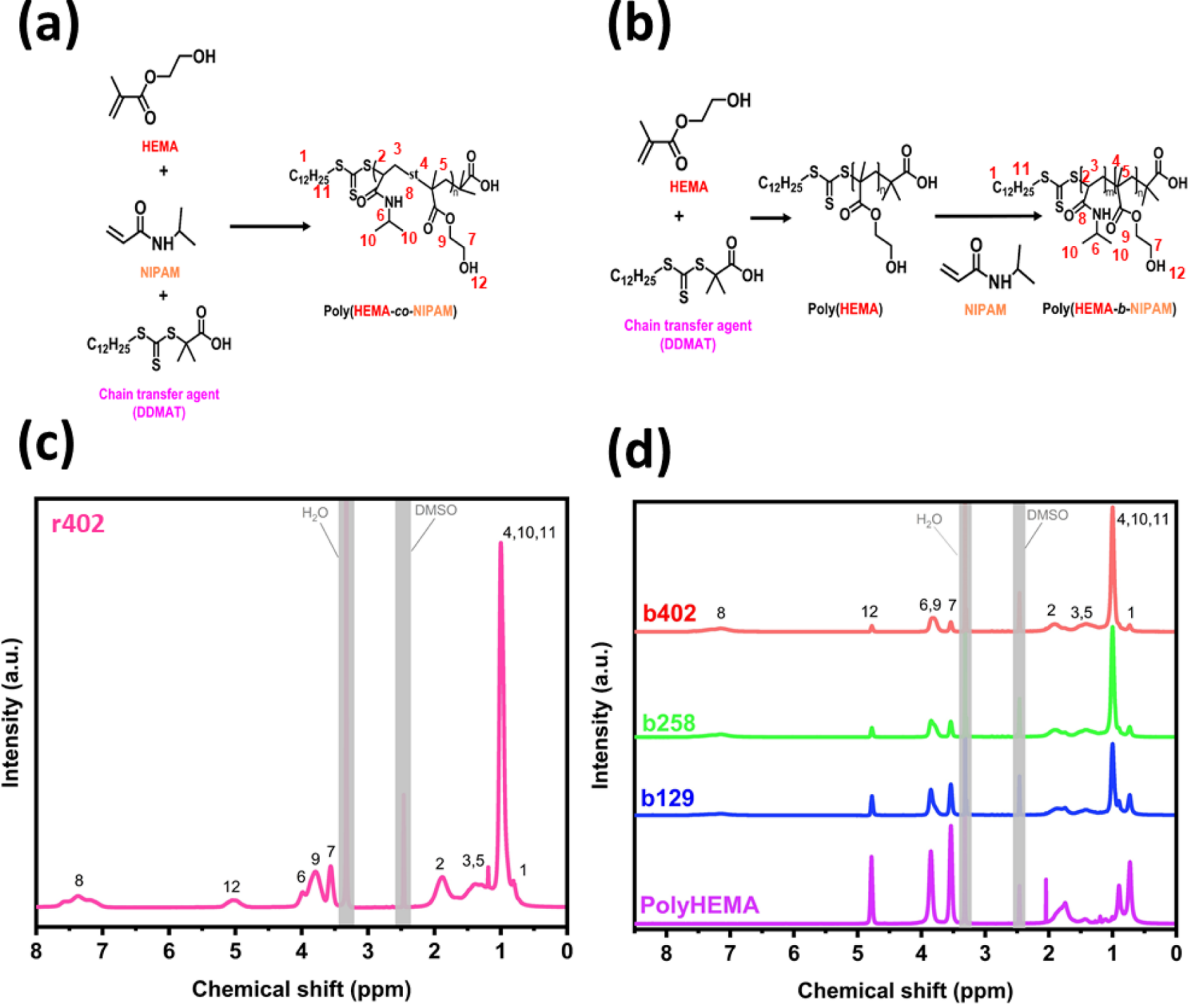
(a) Synthesis of poly­(HEMA-*co*-NIPAM). (b) Synthesis of block copolymer poly­(HEMA-*b*-NIPAM). (c) ^1^H NMR spectrum of the resulting copolymer, r402. (d) ^1^H NMR spectrum of the resulting block copolymer.

### 2.3 Synthesis of RAFT Agent P­(HEMA_44_)

HEMA (7.5 g, 57.63 mmol), DDMAT (480 mg, 1.316 mmol), and AIBN (43.5 mg, 0.265 mmol) were added to a three-necked round-bottom flask and dissolved in 20 mL of anhydrous DMF to achieve a total solid content of 40 wt %. The reaction mixture was degassed under vacuum and purged with nitrogen for 30 min to eliminate dissolved oxygen. Polymerization was then conducted at 70 °C for 16 h under constant stirring at 300 rpm. Upon completion, the reaction was quenched in an ice bath (0 °C), and the crude product was purified by dialysis against methanol for 24 h and deionized water for 48 h. The dialyzed solution was subsequently freeze-dried for 3 days to yield the homopolymer macro-RAFT agent, poly­(HEMA)_44_.^17^

### 2.4 Synthesis of Block Copolymer Poly­(HEMA-*b*-NIPAM)

Block copolymers with varying NIPAM chain lengths were synthesized via chain extension of poly­(HEMA)_44_, using NIPAM as the second monomer (Table 1). As an example, poly­(HEMA)_44_ (0.5 g, 0.048 mmol), NIPAM (0.7 g, 6.18 mmol), and AIBN (2.0 mg, 0.012 mmol) were dissolved in 10 mL of anhydrous DMF in a nitrogen-purged three-necked flask. Following degassing under vacuum and nitrogen purging, the polymerization was carried out at 85 °C for 48 h with continuous stirring at 300 rpm (Figure 1b). The reaction was quenched in an ice bath, and the crude product was purified via dialysis against methanol for 24 h followed by deionized water for 48 h.^18^ Final purification was performed by freeze-drying over 3 days to afford the amphiphilic block copolymer, P­(HEMA-*b*-NIPAM), hereafter referred to as b129.

**Table 1:** Recipe for the Synthesis of Poly­(HEMA-*b*-NIPAM)

sample	sample code	poly(HEMA)_44_ (mmol)	NIPAM (mmol)
P(HEMA_44_-*b*-NIPAM_129_)	b129	0.048	6.18
P(HEMA_44_-*b*-NIPAM_258_)	b258	0.048	12.36
P(HEMA_44_-*b*-NIPAM_402_)	b402	0.048	19.30

### 2.5 Hydrogel Formation via DTPA Cross-Linker

All hydrogels were fabricated using a consistent precursor composition in which the copolymer (0.051 g), DTPA (0.017 g), DMAP (0.0017 g), and EDC.HCl (0.034 g) served as the reactive components. The reagents were dissolved in DMSO, with the solvent volume modulated to tune the solid content-0.136 mL for 40 wt % and 0.170 mL for 50 wt % formulations. The copolymer, DTPA, and DMAP were premixed to ensure uniform dispersion, after which the solution was combined with the EDC.HCl activator. This immediate introduction triggered rapid carboxyl activation and subsequent ester bond formation with HEMA-based hydroxyl groups.^19^ The random copolymer (r402) hydrogel solidified within ∼5–10 s, whereas the block copolymer hydrogels exhibited slightly slower gelation, completing network formation within ∼12–15 s under identical reaction conditions. The freshly formed networks were left undisturbed for 1 h to allow structural stabilization before further characterization.

### 2.6 Swelling and Water-Uptake Measurement

The equilibrium swelling behavior of the hydrogels was evaluated in deionized water at room temperature. Preformed hydrogels were cut into identical dimensions and dried to constant weight under vacuum to obtain the dry mass (*W*_d_). The dried samples were then immersed in excess deionized water and allowed to swell until equilibrium was reached (24 h).^20^ The swollen hydrogels were gently blotted to remove surface water and weighed to obtain the swollen mass (*W*_s_). The swelling ratio (SR) was calculated using the following equation

$$swelling\;ratio\;\left(SR\right)=(W_{s}{-W}_{d}{)/W}_{d}$$

All measurements were performed in triplicate, and the average values are reported. Detailed explanations were discussed in Supporting Information.

### 2.7 Structural Characterization

The structural composition of the synthesized copolymers P­(HEMA-*co*-NIPAM) and P­(HEMA-*b*-NIPAM) was confirmed by proton nuclear magnetic resonance (^1^H NMR) spectroscopy. Approximately 30 mg of each polymer sample was dissolved in 0.6 mL of DMSO-*d*_6_, and spectra were recorded using a 400 MHz NMR spectrometer. The obtained spectra were used to verify the successful incorporation of monomers and to determine their integration ratios. Functional group analysis was performed using Fourier-transform infrared spectroscopy (FTIR). Prior to pellet preparation, potassium bromide (KBr) powder and the polymer samples were dried at 120 °C overnight to eliminate moisture. The dried components were then mixed in a 1:6 weight ratio and finely ground into a homogeneous powder. The mixture was compressed into translucent discs using a manual pellet press. FTIR spectra were recorded in the range of 4000–400 cm^–1^, with a pure KBr pellet used to obtain the background spectrum. Thermal degradation behavior of the polymers was assessed by thermogravimetric analysis (TGA). Approximately 10 mg of each freeze-dried sample, poly­(HEMA)/DTPA, r402/DTPA, b129/DTPA, b258/DTPA, and b402/DTPA, was evenly distributed onto platinum pans. The samples were heated from room temperature to 900 °C at a rate of 10 °C/min under a nitrogen atmosphere. The glass transition temperature (*T*_g_) of the polymers was determined using differential scanning calorimetry (DSC). Roughly 10 mg of each polymer was analyzed under a nitrogen atmosphere. Heating was carried out from room temperature to either 230 or 250 °C at a rate of 20 °C/min, depending on the polymer’s composition.

### 2.8 Mechanical and Morphological Studies

For scanning electron microscopy (SEM) analysis, hydrogel samples were freeze-dried 1 day prior to imaging to eliminate residual moisture and preserve structural integrity. The dried specimens were mounted onto carbon adhesive tabs and sputter-coated with a thin layer of platinum to prevent surface charging during imaging. Prior to insertion into the SEM chamber, the mounted samples were further air-dried using a gentle nitrogen stream to ensure complete dryness and avoid instrument contamination. The surface morphology and internal porous structures of the hydrogels were then visualized using SEM. Rheological measurements were performed using a rotational rheometer to evaluate the viscoelastic and mechanical behavior of the hydrogels. Rheological measurements were performed using an Anton Paar MCR 302 rheometer equipped with a parallel-plate geometry (25 mm diameter, 1 mm gap) at 25 °C. Formulations with solid contents of 40 and 50 wt % were prepared in 3 mL plastic syringes, each containing a final volume of 1 mL. For the strain sweep analysis, strain was varied from 0.1% to 1000% at a constant angular frequency of 1 Hz over a time span of 500 s. In the frequency sweep analysis, frequency ranged from 1 to 100 Hz at a fixed strain of 0.1%.^21^ The storage modulus (*G*′) and loss modulus (*G*″) were extracted from the data and plotted using Origin software to characterize the gel’s viscoelastic profile. Additionally, viscosity measurements were conducted on polymer solutions using a rotational rheometer in steady-shear mode at 25 °C. Apparent viscosity was recorded as a function of shear rate, and representative values at low shear were used for comparison to minimize shear-thinning effects. Measurements were performed for both random and block copolymers under identical conditions. Further details were explained in Supporting Information.

### 2.9 Gelation and Redox Responsive Properties

Hydrogels were prepared at varying solid contents (10, 20, 30, 40, and 50 wt %) in 1.5 mL microcentrifuge tubes to evaluate critical gelation thresholds. After allowing the mixtures to rest undisturbed at room temperature, each tube was inverted to assess flow behavior.^22^ Gelation was considered successful when the hydrogel resisted flow upon inversion, which was observed at 40 and 50 wt %. For self-healing analysis, two identical hydrogel samples from each composition (r402/DTPA, b129/DTPA, b258/DTPA, and b402/DTPA) were prepared, with contrasting-colored dyes incorporated to enable visual differentiation. Each hydrogel was cut cleanly in half, and opposing halves were immediately brought into contact and allowed to stand at room temperature without external stimuli. Healing progression was documented photographically at defined time intervals. Successful healing was confirmed when the hydrogel could be lifted with tweezers without visible separation at the interface, indicating mechanical reintegration. To assess the redox responsiveness of the hydrogels, 10 μL of freshly prepared DTT solution was applied to the surface of each formulation (r402/DTPA, b129/DTPA, b258/DTPA, and b402/DTPA) to trigger disulfide bond cleavage and induce the gel–sol transition. Complete liquefaction typically occurred within 5–10 min. Subsequently, an equal volume of H_2_O_2_ was added to oxidize the liberated thiol groups, enabling disulfide bond reformation and restoring the gel state, which was achieved within 15 min. This two-step redox cycling process demonstrated the reversible redox-triggered sol–gel transitions of hydrogels and their potential for intelligent, stimulus-driven applications.^23^ Self-healing behavior was investigated using oscillatory step-strain rheology. Measurements were conducted at 25 °C using a parallel-plate geometry. The hydrogels were subjected to alternating low strain (0.1%, within the linear viscoelastic region) and high strain (500%, beyond the yield point) at a constant angular frequency. Each strain interval was maintained for a fixed duration, and the evolution of storage modulus (*G*′) and loss modulus (*G*″) was continuously recorded to assess network disruption and recovery.

## 3 Results and Discussion

### 3.1 Structural Analysis of the Poly­(HEMA-*co*-NIPAM) and Poly­(HEMA-*b*-NIPAM) Copolymers

The ^1^H NMR spectrum of P­(HEMA-*co*-NIPAM) dissolved in DMSO-*d*_6_ exhibits a characteristic solvent signal at 2.50 ppm (Figure 1c). A distinct resonance at approximately 1.00 ppm corresponds to the methyl protons of the chain transfer agent (DDMAT), confirming the presence of terminal RAFT functionality. The region between 0.90 and 2.00 ppm displays multiple overlapping signals arising from the isopropyl groups of NIPAM, methyl groups of HEMA, and the methylene (−CH_2_−) and methine (−CH−) protons along the polymer backbone. The methylene protons on the HEMA side chain are observed at 3.56 ppm and were used as the internal reference (integral set to 2). In addition, the hydroxyl proton of HEMA appears at 5.04 ppm, while the amide proton of NIPAM is detected at 7.36 ppm, both confirming the incorporation of respective monomer units into the copolymer chain. Integration analysis of these signals yielded an approximate NIPAM to HEMA molar ratio of 3:1, corresponding to a segmental composition of NIPAM_154_:HEMA_53_. This result indicates successful copolymerization and reasonably efficient incorporation of both monomers under the selected RAFT conditions.

The ^1^H NMR spectra of the block copolymers (b129, b258, b402) confirmed the presence of both HEMA and NIPAM segments. For instance, in b258, the methylene protons of the HEMA side chains appeared at 3.54 ppm (2H), while overlapping peaks at 3.82 ppm corresponded to methine and methylene protons from both HEMA and NIPAM blocks (Figure 1d). Integration analysis indicated an approximate NIPAM/HEMA molar ratio of 3:1 in b258, lower than the theoretical feed ratio, suggesting incomplete chain extension or lower NIPAM reactivity during the second polymerization step. These compositional differences are likely a contributing factor to the observed variations in cross-linking density, mechanical performance, and self-healing efficiency between random and block copolymer hydrogels. Table 2 provides a detailed comparison of theoretical feed ratios and final copolymer compositions for all samples, highlighting the importance of NMR in verifying actual polymer structure. Although these data allow estimation of monomer incorporation, accurate molecular weight determination from ^1^H NMR was not feasible because the DDMAT RAFT end-group protons overlap with HEMA backbone signals. Comparisons of polymer architecture, rheology, and gelation behavior are used to interpret differences between random and block copolymer hydrogels.

**Table 2:** Molar Ratios of NIPAM to HEMA Feed Ratio and ^1^H NMR Results in Block Copolymer

	(NIPAM/HEMA)^Feed^	(NIPAM/HEMA)^NMR^
name	(mole)	(mole)
b129	3/1	1/1
b258	6/1	3/1
b402	9/1	6/1

### 3.2 Functional Group Analysis

The FTIR spectra of poly­(HEMA), P­(NIPAM), and the synthesized copolymers (r402, b129, b258, b402) are shown in Figure 2a. Broad absorption bands observed between 3280 and 3500 cm^–1^ are attributed to stretching vibrations of hydroxyl (−OH) and secondary amide (−NH) groups, indicating the hydrophilic nature of both monomer units.^24^ A sharp absorption peak at 1726 cm^–1^ corresponds to the ester carbonyl stretching vibration from the HEMA component, confirming successful polymerization. In addition, the presence of characteristic amide I and amide II bands at 1660 cm^–1^ and 1550 cm^–1^, respectively, verifies the incorporation of NIPAM units. Notably, a gradual increase in the intensity of these amide peaks correlates with increasing NIPAM content in the copolymer series, particularly in b258 and b402, thus supporting the ^1^H NMR findings regarding segmental composition. The FTIR spectra of DTPA-cross-linked hydrogels (r402/DTPA, b129/DTPA, b258/DTPA, b402/DTPA) are also represented in Figure 2b. A new absorption band emerging around 512 cm^–1^ is assigned to S–S stretching vibrations, indicating the successful incorporation of disulfide bonds through the cross-linkage of DTPA. Additionally, a further enhancement of the ester carbonyl peak at 1726 cm^–1^ suggests covalent bond formation between the hydroxyl groups of HEMA and the carboxyl groups of DTPA, forming ester linkages within the hydrogel network.^25^ These spectral signatures collectively confirm the structural integrity of the redox-responsive hydrogel system, validate successful copolymerization and cross-linking, and highlight the tunable nature of functional group distribution via composition control.

**Figure 2:**
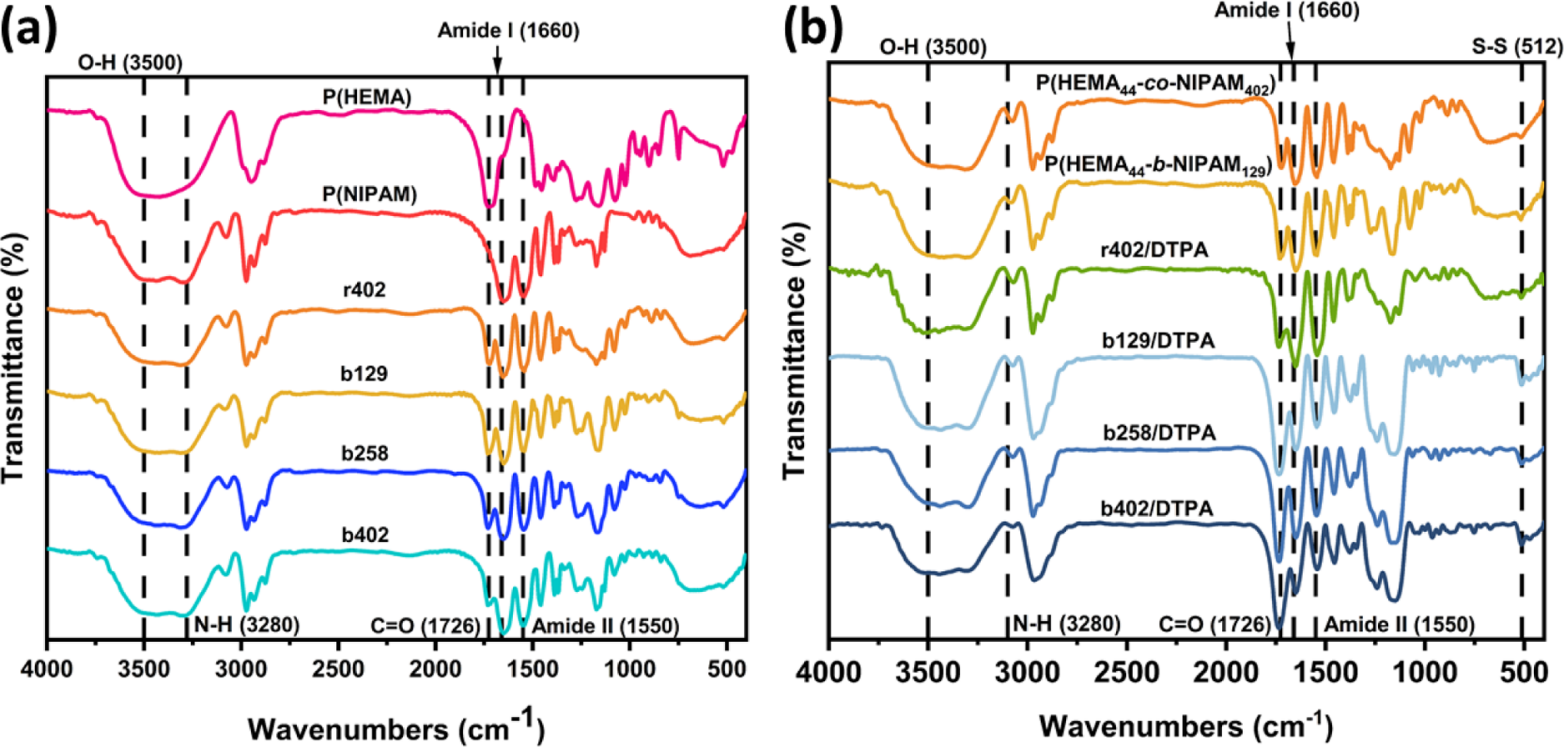
(a) FTIR spectra of individual homopolymers poly­(HEMA), P­(NIPAM), and synthesized copolymers (r402, b129, b258, b402), (b) FTIR spectra of DTPA-cross-linked hydrogels.

### 3.3 Gelation behavior

Figure 3 represents the gelation behavior of r402/DTPA (Figure 3a) and block copolymer-based hydrogels (b129/DTPA, b258/DTPA, and b402/DTPA) (Figure 3b–d) at varying solid contents (10–50 wt %). As expected, increasing the polymer concentration significantly enhanced both the rate and robustness of gel formation. At lower concentrations (≤30 wt %), incomplete or delayed gelation was observed, particularly in block copolymer systems, due to insufficient chain overlap and limited cross-link density. In contrast, at 40 and 50 wt %, all hydrogel formulations formed stable, self-supporting gels. This behavior is primarily attributed to the higher density of polymer chains at increased concentrations, which facilitates more frequent intermolecular interactions-including hydrogen bonding, van der Waals forces, and covalent ester cross-linking. These interactions contribute to the development of a denser and more resilient three-dimensional polymeric network. Notably, r402/DTPA exhibited more rapid and uniform gelation than its block copolymer counterparts, suggesting that random copolymer architecture supports a more homogeneous distribution of reactive functional groups throughout the matrix.

**Figure 3:**
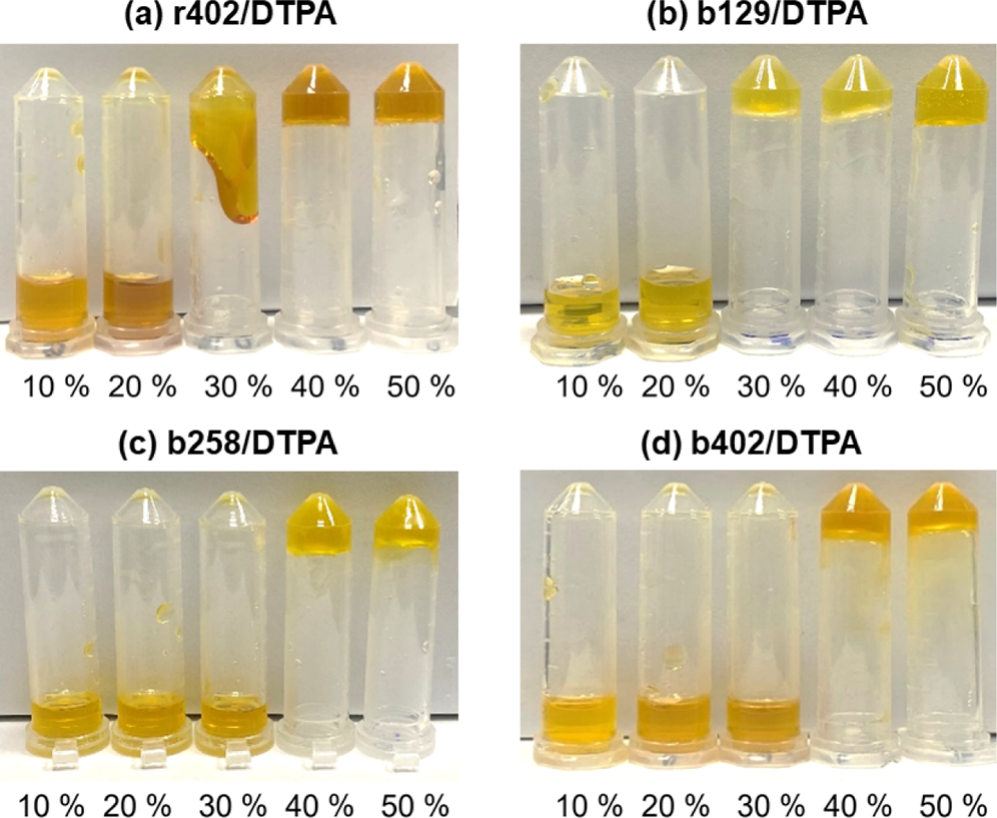
Visual observation of gelation test at various solid contents (10–50 wt %), confirming gel formation at ≥ 40 wt %.

### 3.4 Thermal Properties

Thermogravimetric analysis (TGA) and derivative thermogravimetry (DTG) curves are shown in Figure 4. The data reveal that block copolymers with longer NIPAM blocks, particularly b258 and b402, exhibit a distinct two-step thermal degradation profile, in contrast to the single-step degradation observed in poly­(HEMA) and r402. This two-stage degradation reflects the presence of phase-separated segments with different thermal stabilities, a typical feature in block copolymer systems.

**Figure 4:**
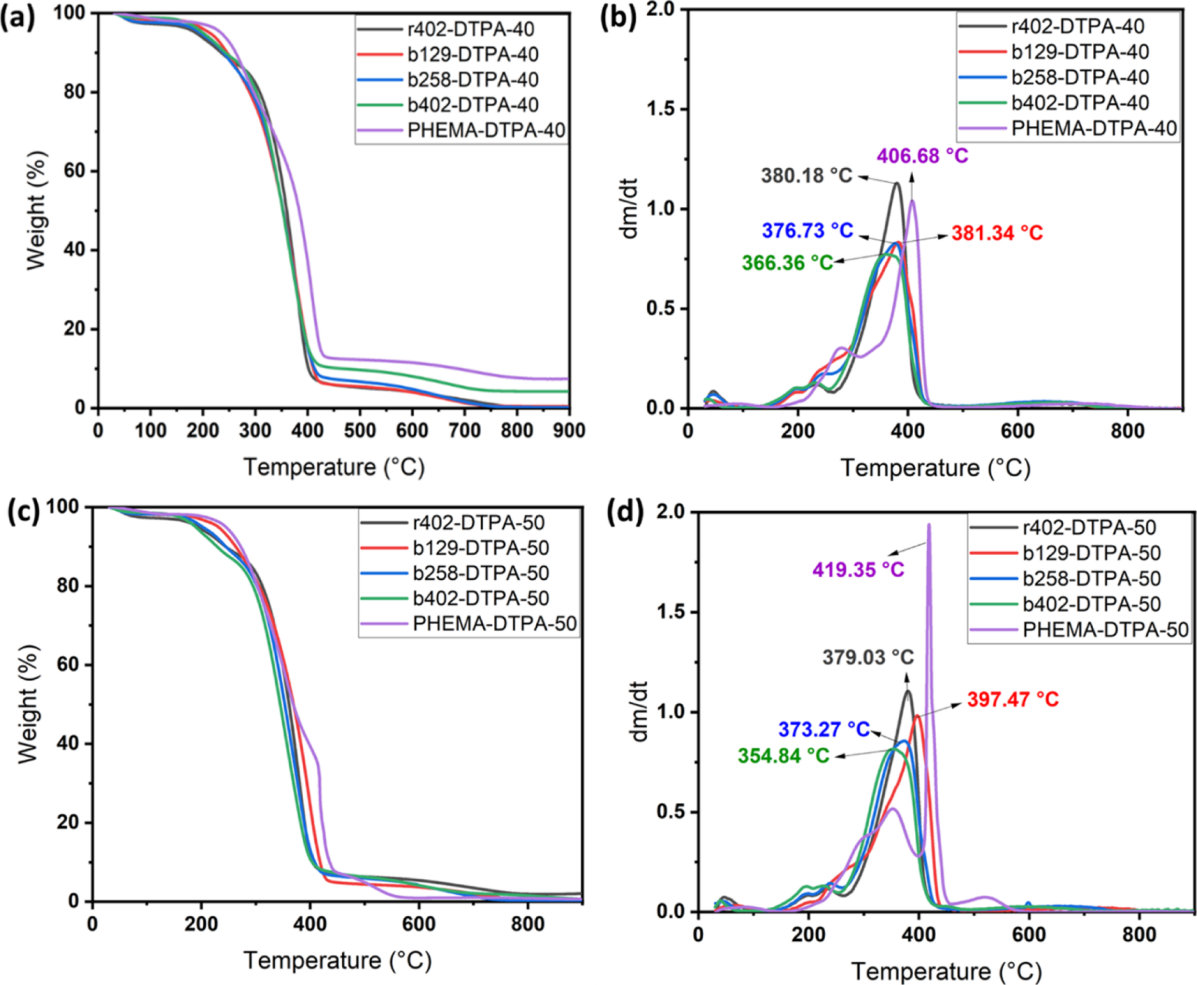
TGA (a,c) and DTG (b,d) analyses of hydrogels, highlighting the effect of macromolecular architecture and solid content on thermal degradation behavior and stability.

The thermal behavior of b129 closely resembles that of poly­(HEMA), indicating a lower degree of NIPAM incorporation, consistent with ^1^H NMR integration and cloud point measurements. Among all samples, poly­(HEMA) exhibited the highest maximum degradation temperature, highlighting its superior thermal stability, likely due to its compact, hydrogen-bonded hydroxyl-rich structure (Figure 4a,c). As the NIPAM content increased, the maximum degradation temperature decreased, most notably in b402, suggesting that higher NIPAM content compromises thermal robustness, possibly due to the presence of more thermally labile isopropylamide segments. Interestingly, r402/DTPA hydrogels demonstrated higher thermal resistance than their block copolymer counterparts (e.g., b402/DTPA) at both 40 and 50 wt % solid content (Figure 4b,d). This suggests that random copolymer networks possess intrinsically greater thermal stability, likely due to more homogeneous monomer distribution and uniform cross-linking density throughout the hydrogel matrix. The DSC thermograms of poly­(HEMA), P­(NIPAM), r402, b129, b258, and b402 are shown in Figure S1. The measured glass transition temperatures (*T*_g_) were as follows: poly­(HEMA) −85.4 °C; P­(NIPAM) −134.8 °C; r402–135.7 °C; b129–126.3 °C; b258–125.2 °C; and b402–124.8 °C. The high *T*_g_ values of P­(NIPAM) and r402 reflect the rigid, hydrogen-bonded nature of the NIPAM segments and their influence on chain mobility. Notably, r402 exhibited the highest *T*_g_ among all copolymers, despite its lower NIPAM content compared to b402. This suggests that random incorporation of NIPAM may restrict segmental motion more effectively than block architectures. A clear trend was observed in block copolymers: *T*_g_ decreased with increasing NIPAM block length. This is attributed to the flexibility imparted by the isopropyl and amide groups of NIPAM, which act as internal plasticizers. Additionally, longer NIPAM blocks may reduce cross-linking density and restrict interchain interactions, further lowering *T*_g_ due to decreased entanglement and reduced network stiffness.

### 3.5 Morphological Analysis

Figure 5 represents SEM micrographs of the freeze-dried hydrogel surfaces. r402/DTPA hydrogels exhibited a uniform and interconnected porous morphology, indicative of a homogeneous and statistically random cross-linking pattern throughout the polymer matrix (Figure 5a,e). This structure reflects the even distribution of HEMA and NIPAM units in the random copolymer, which promotes uniform network formation and consistent porosity. In contrast, b402/DTPA hydrogels displayed irregular and heterogeneous pore architectures, characterized by discontinuous and anisotropic domains (Figure 5d,h).

**Figure 5:**
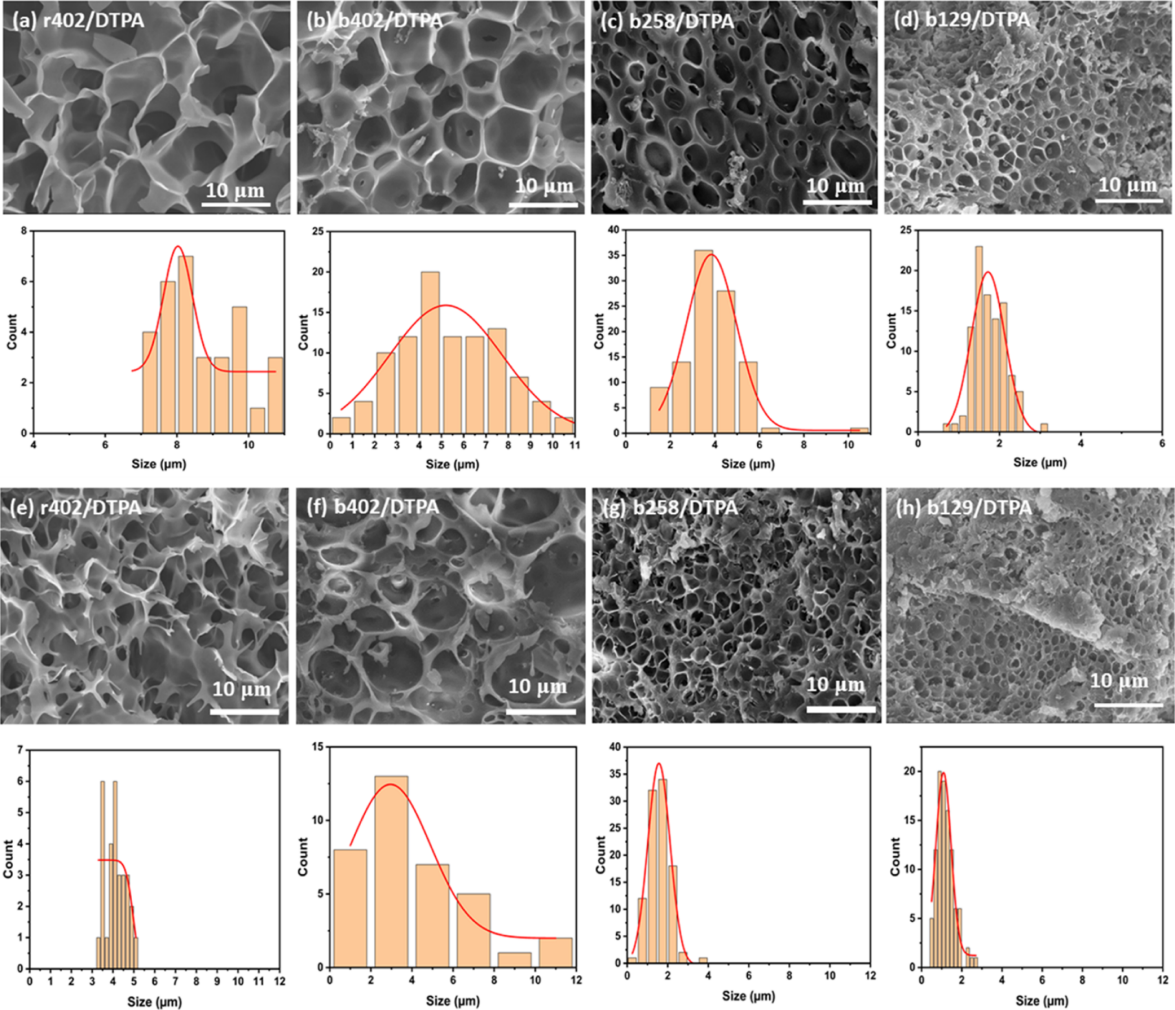
SEM micrographs of freeze-dried hydrogels at (a–d) 40 wt % and (e–h) 50 wt % solid content: (a,e) r402/DTPA, (b,f) b129/DTPA, (c,g) b258/DTPA, and (d,h) b402/DTPA.

This is attributed to the microphase separation inherent in block copolymer systems, where only the HEMA segments participate in covalent cross-linking via DTPA, while the non-cross-linkable NIPAM blocks form flexible, inert domains. These segregated domains introduce structural inhomogeneity and reduce the overall network integrity. Further comparisons among b129/DTPA (Figure 5b,f), b258/DTPA (Figure 5c,g), and b402/DTPA clearly demonstrate that increasing NIPAM block length leads to larger pore sizes and decreased cross-linking density. Longer NIPAM segments not only dilute the concentration of cross-linkable HEMA units but also create entropic barriers that hinder effective network formation, thereby producing more open, less densely packed structures. At a higher solid content of 50 wt %, all hydrogel samples exhibited notably smaller pore diameters relative to their 40 wt % counterparts. This trend is likely due to reduced solvent availability and mobility during gelation and subsequent lyophilization, which constrains pore growth and results in more compact network structures.

### 3.6 Rheological behavior and Mechanical Properties of the Hydrogels

The rheological performance of the hydrogels was evaluated via strain and frequency sweep tests. Figure 6 shows the storage modulus (*G*′) and loss modulus (*G*″) as functions of strain for hydrogels with varying compositions and solid contents. All samples exhibited solid-like behavior at low strain amplitudes, where *G*′ consistently exceeded *G*″, indicating the formation of stable, elastic gel networks.^26^ Among the formulations, r402/DTPA displayed the highest critical strain before undergoing gel–sol transition, with a fracture strain of 295.9% at 40 wt %, in contrast to b402/DTPA, which fractured at only 99.0% (Figure 6a,b). This significant difference indicates that random copolymer hydrogels possess a more homogeneous and densely cross-linked network, allowing them to withstand greater mechanical deformation before failure. At 50 wt %, the mechanical strength of all hydrogels improved, attributed to the higher polymer concentration, which increases the number of available reactive sites for cross-linking. This leads to tighter, more interconnected network structures, thereby enhancing gel stiffness and toughness. The frequency sweep results further support this trend (Figure S2). Across the tested frequency range (1–100 Hz), *G*′ remained consistently greater than *G*″ in all samples, confirming the dominance of elastic behavior over viscous dissipation. Hydrogels with higher solid content exhibited elevated *G*′ values, indicating stronger viscoelastic frameworks. Among the block copolymers, b402/DTPA showed the lowest mechanical strength, primarily due to its lower HEMA content, which limits the availability of cross-linkable –OH groups. Since the cross-linking reaction is governed by interactions between the hydroxyl groups of HEMA and the carboxyl groups of DTPA, a reduction in HEMA content proportionally reduces the cross-linking density. These findings are corroborated by SEM images, which reveal larger, less organized pores in b402 hydrogels compared to r402. Figure 6c,d compare the viscoelastic behavior of b129/DTPA, b258/DTPA, and b402/DTPA hydrogels, each containing different NIPAM block lengths. Among these, b402/DTPA consistently exhibited the lowest *G*′, reinforcing the inverse relationship between NIPAM content and mechanical robustness. The cross-linking mechanism is dominated by the HEMA segment, and as NIPAM blocks grow longer, the dilution of HEMA reduces the extent of network formation. Interestingly, the mechanical properties of b129/DTPA and b258/DTPA were relatively similar, despite differences in NIPAM block length. This may be attributed to nonuniform network formation or kinetic limitations during hydrogel blending and cross-linking, which could offset expected structural differences. The viscoelastic properties of un-cross-linked polymer formulations were examined to establish the role of DTPA-mediated covalent cross-linking. In the absence of DTPA, all polymer systems displayed liquid-like behavior, characterized by *G*″ values consistently higher than *G*′ across the tested frequency range. No *G*′/*G*″ crossover was observed, indicating the absence of a percolated network (Figure S4). In contrast, DTPA-cross-linked hydrogels exhibited solid-like behavior with *G*′ exceeding *G*″ by more than an order of magnitude, confirming the formation of a stable three-dimensional network. This trend is consistent with previous observations, where higher solid content enables denser cross-linking, leading to improved mechanical integrity and resistance to deformation.

**Figure 6:**
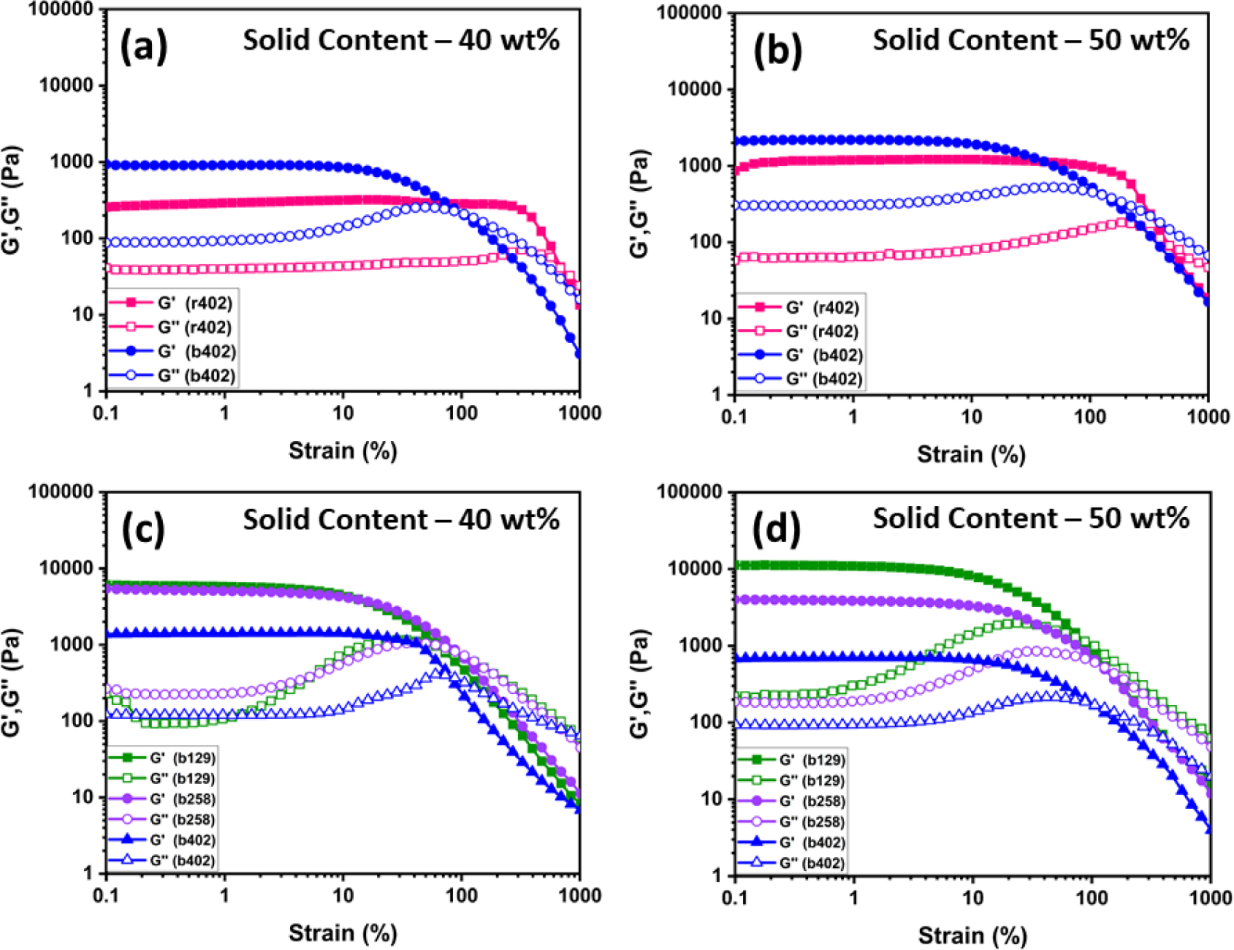
Strain-dependent rheological profiles of hydrogels at different solid contents (a) r402/DTPA, (b) b129/DTPA, (c) b258/DTPA, (d) b402/DTPA.

### 3.7 Self-Healing, and Redox Responsiveness of Hydrogels

The self-healing capabilities of the hydrogels were visually assessed by bringing color-dyed hydrogel halves into direct contact and monitoring fusion over 24 h (Figure 7). r402/DTPA exhibited rapid and complete self-repair: the junction between the two-halves became indistinguishable, and the healed gel could be lifted without rupture, indicating efficient network reconstruction. Quantitative analysis of mechanical recovery revealed that r402/DTPA regained >90% of its original fracture strain (∼295%), demonstrating strong adhesion and cohesive strength at the interface. Among the block copolymers, b402/DTPA showed relatively better healing, with 50–70% recovery of original fracture strain, likely due to enhanced chain mobility from longer NIPAM segments facilitating bond reformation. In contrast, b129 and b258 hydrogels exhibited lower recovery, reflecting reduced homogeneity and fewer accessible cross-linking sites.^27^ Overall, these results confirm that random copolymer hydrogels possess superior self-healing efficiency and interfacial strength compared to block copolymer networks, correlating with their denser and more uniform cross-linked structure.

**Figure 7:**
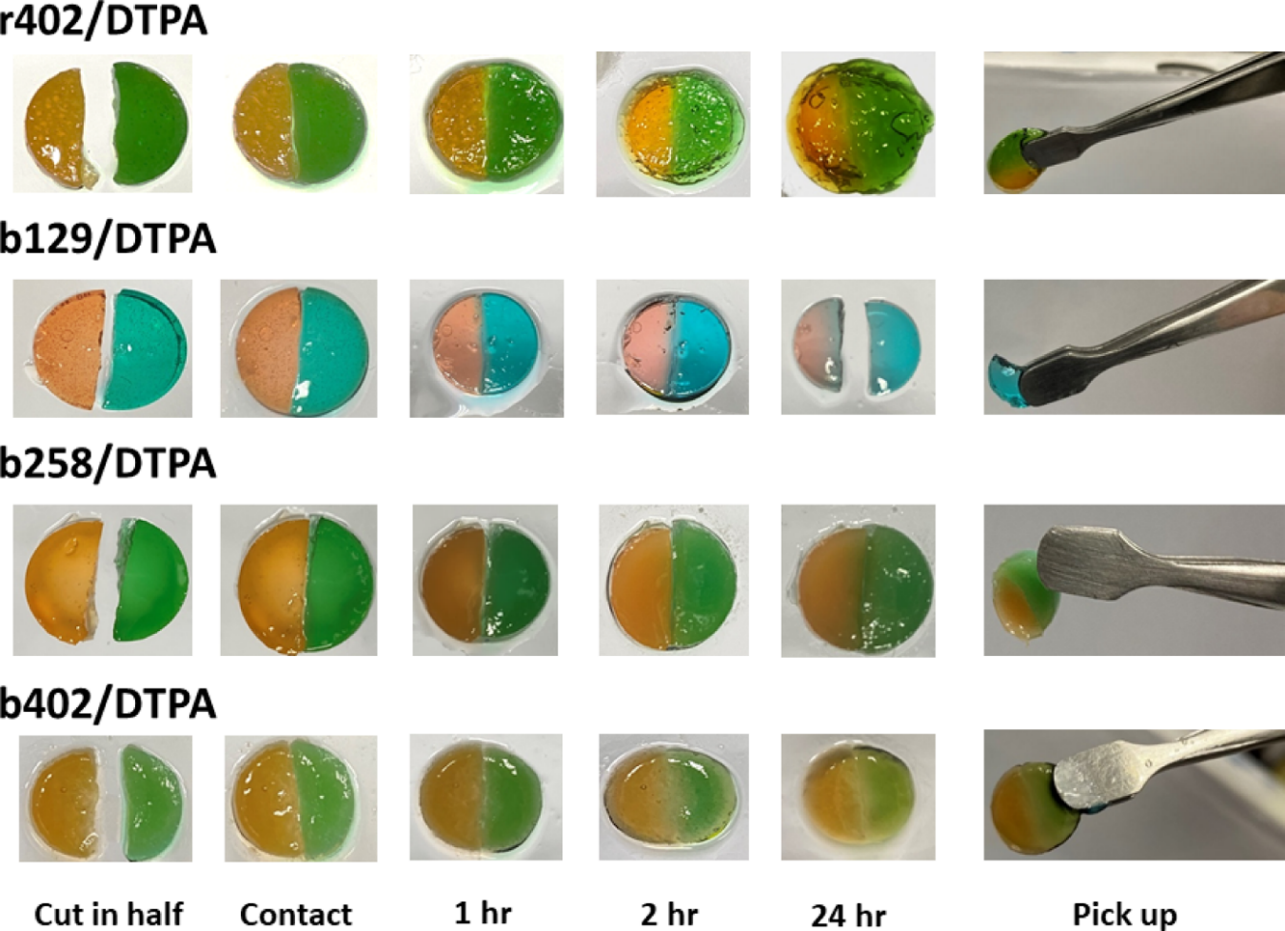
Visual representation of self-healing assessment using dual-colored hydrogels at 40 wt % solid content under room temperature.

Step-strain oscillatory rheology was employed to investigate the self-healing behavior of the hydrogels at the molecular network level. Under low strain (0.1%), all hydrogels exhibited dominant elastic behavior with *G*′ significantly exceeding *G*″, confirming the integrity of the cross-linked network. Upon application of a high strain (500%), *G*′ dropped sharply below *G*″, indicating network rupture and transition to a fluid-like state.^28^ When the strain was subsequently returned to 1%, rapid recovery of *G*′ was observed, demonstrating spontaneous reformation of the polymer network (Figure 8). Notably, the r402/DTPA hydrogel exhibited nearly complete recovery of its initial *G*′ value within a short time, highlighting its efficient self-healing capability (Figure 8A). In contrast, block copolymer hydrogels showed partial and slower modulus recovery, consistent with their lower cross-link density and heterogeneous network architecture (Figure 8B–D). These results confirm that self-healing in the hydrogels originates from reversible disulfide bond reformation rather than simple physical chain entanglement, providing direct rheological evidence of dynamic covalent network reconstruction.^29^

**Figure 8:**
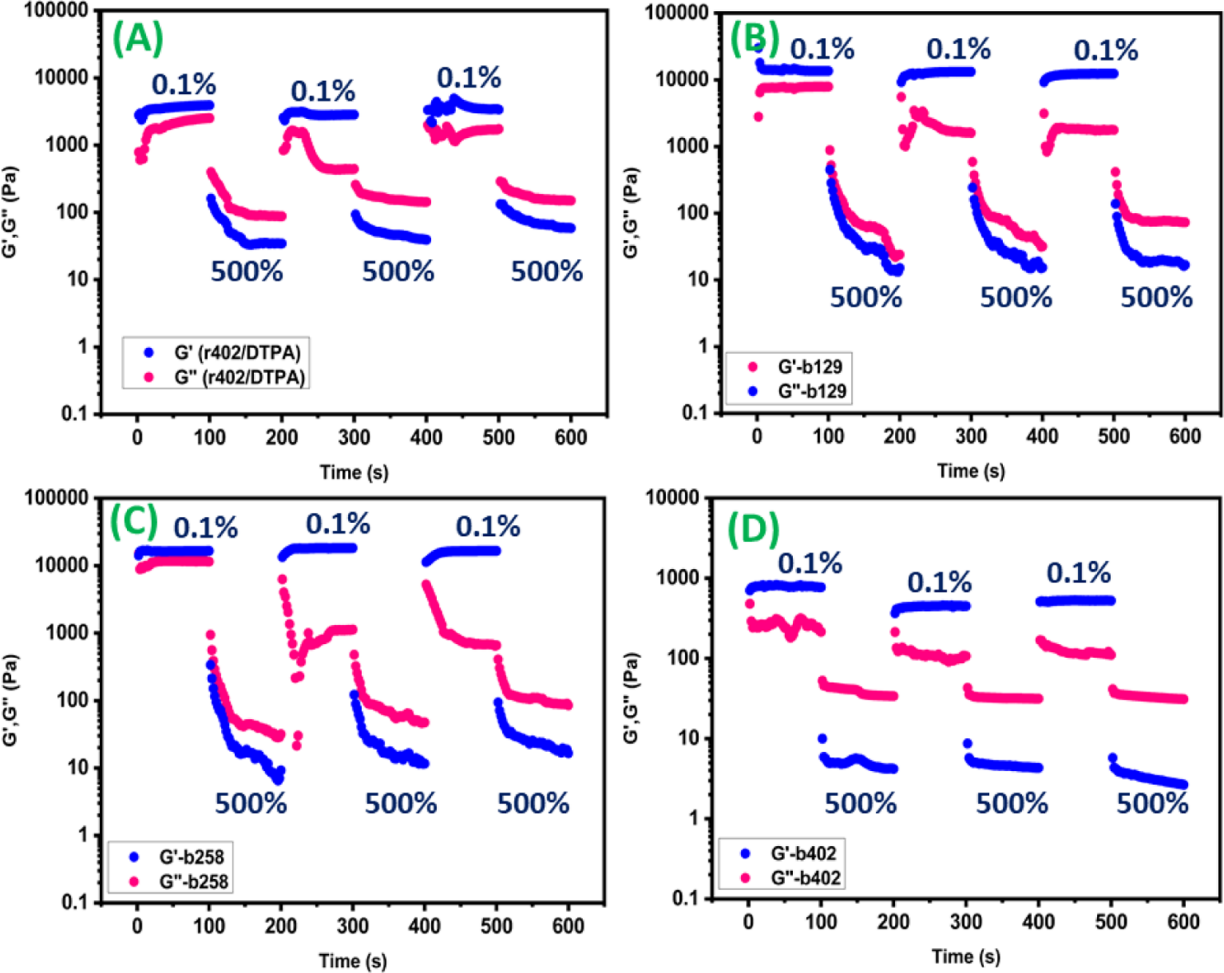
Step-strain oscillatory rheological analysis demonstrating the self-healing behavior of DTPA-cross-linked hydrogels.

To investigate redox responsiveness, the hydrogels were first treated with 10 μL of 10 wt % DTT, inducing a gel-to-sol transition within 5–10 min through cleavage of disulfide bonds into thiol groups (Figure 9). Subsequent addition of 10 μL of H_2_O_2_ reoxidized the thiols, facilitating disulfide bond reformation and restoring the gel structure within 15 min. This demonstrates the reversible sol–gel transition of all hydrogel formulations. Among them, r402/DTPA consistently regenerated into a mechanically robust gel, highlighting its superior network integrity and redox responsiveness (Figure 9a). The b402/DTPA hydrogel also reformed, but its partial collapse indicates lower cross-link density, limiting full structural recovery (Figure 9d). Notably, b129/DTPA (Figure 9b) exhibited better redox recovery than b258 (Figure 9c) and b402, likely due to its higher HEMA content, which provides more –OH groups for DTPA-mediated cross-linking and a more cohesive network capable of dynamic reassembly.^30^

**Figure 9:**
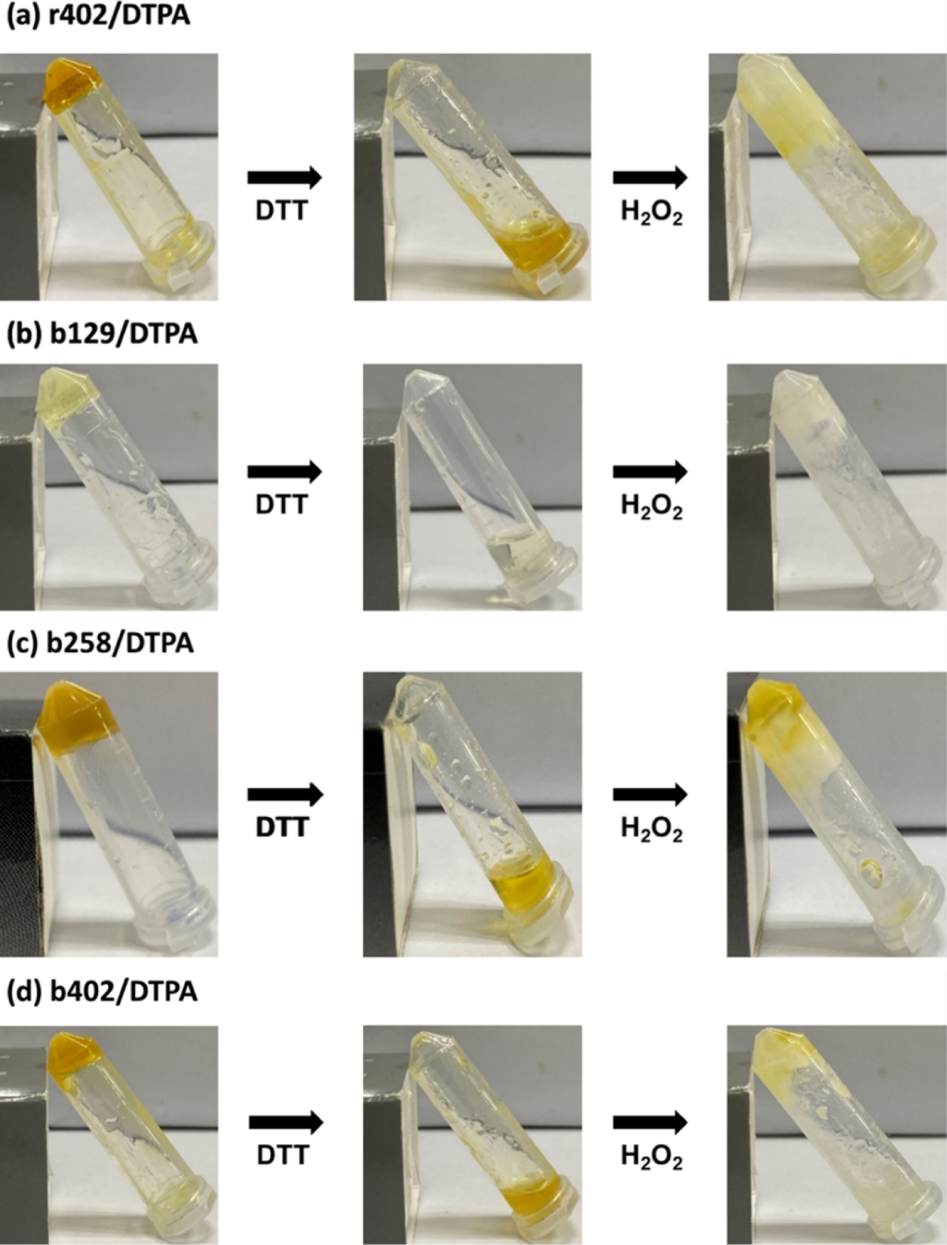
Photographs of r402/DTPA (a), b129/DTPA (b), b258/DTPA (c), and b402/DTPA (d) hydrogels subjected to sequential redox responsive sol–gel transitions of hydrogels under reductive and oxidative conditions.

## 4 Conclusions

In conclusion, our study systematically elucidates the decisive impact of polymer architecture on the performance of redox-responsive copolymer hydrogels. Random copolymer networks, constructed from poly­(HEMA-*co*-NIPAM) and cross-linked with DTPA, demonstrate rapid gelation (≤30 s), high elasticity (fracture strain: 295.9% at 40 wt %), and remarkable thermal stability (maximum degradation temperature: 380 °C; glass transition: 135 °C). Morphological analysis reveals homogeneous pore structures (20–25 μm) that contrast sharply with the irregular, larger pores (35–40 μm) seen in block copolymer hydrogels. Mechanically, the random networks also deliver greater resilience and self-healing, enabled by a denser cross-link distribution and dynamic disulfide bonds. In contrast, block copolymer hydrogels (poly­(HEMA-*b*-NIPAM)) display slower gelation, lower toughness (fracture strain: 99.0% at 40 wt %), reduced thermal resistance (degradation at 340 °C; *T*_g_ 125 °C), and less efficient self-repair. These data highlight the critical importance of random copolymer architecture for engineering hydrogels with optimal mechanical, thermal, and adaptive properties, providing a robust foundation for the rational design of next-generation materials in smart wound healing and controlled drug delivery.

## Supplementary Material



## Notes

Includes DSC thermograms, swelling/water-uptake analysis, viscosity studies, and additional rheological measurements of the hydrogels (PDF)
