# Supplementary material for: Architectural Tuning of Redox-Responsive Copolymer Hydrogels: Fast Gelation, Self-Healing, and Superior Mechanics via Block vs Random Networks
Source: ACS Polym Au. 2026 Feb 10;6(2):575–86. doi: 10.1021/acspolymersau.5c00166 (PMC13067162; doi:10.1021/acspolymersau.5c00166)
Supplement: Supplementary file 1 [file lg5c00166_si_001.pdf]

Architectural Tuning of Redox-Responsive Copolymer Hydrogels: Fast Gelation, Self-Healing, and Superior Mechanics via Block vs. Random Networks

Dhayanithi Senthilkumar[1,2]†, Yun-Jie Liao[1]†, Shr-Shiang Weng[3], Chih-Yu Kuo[1,2,3,4]*

[1]Department of Chemical Engineering and Biotechnology, National Taipei University of Technology, Taipei 10608, Taiwan.

[2]International Graduate Program of Energy and Optoelectronic Materials Program (EOMP), National Taipei University of Technology, Taipei City 10608, Taiwan.

[3]Institute of Biochemical and Biomedical Engineering, National Taipei University of Technology, Taipei City 10608, Taiwan.

[4]High-Value Biomaterials Research and Commercialization Center, National Taipei University of Technology (Taipei Tech), Taipei 10608, Taiwan.

†stands for equal contribution

*Corresponding authors: **Chih-Yu Kuo,** e-mail: chihyukuo@ntut.edu.tw

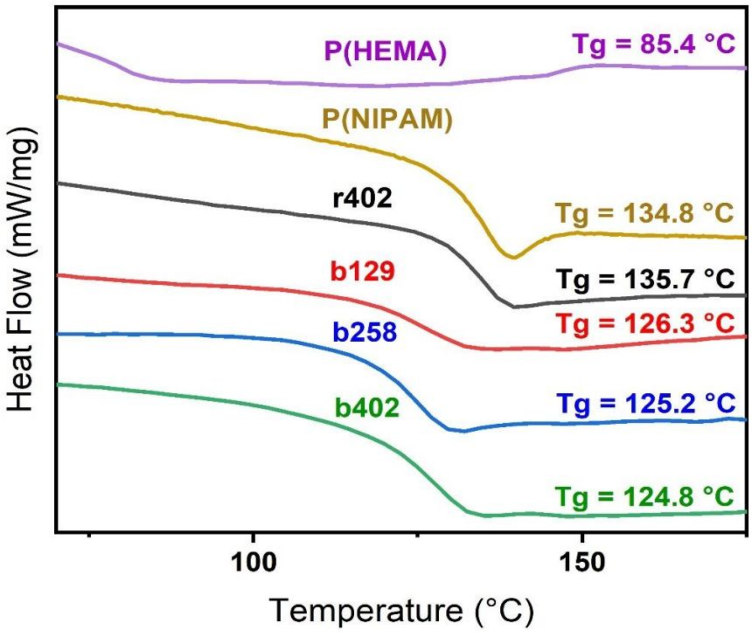

Fig. S1 DSC thermograms of P(HEMA), P(NIPAM), r402, b129, b258, and b402, showing glass transition temperatures (T_g) and illustrating the influence of copolymer architecture and NIPAM content on thermal transitions.

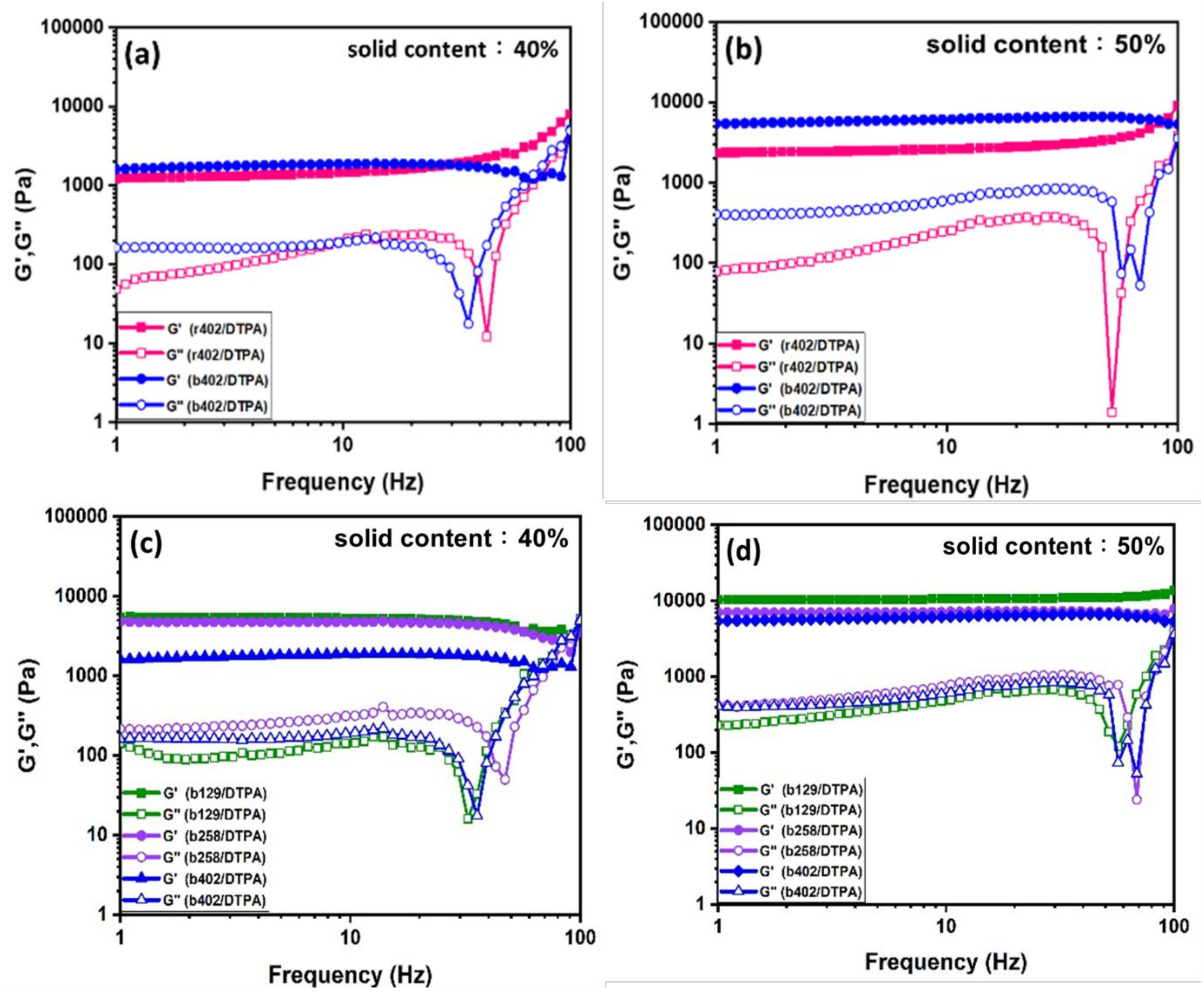

Fig. S2 Frequency-dependent storage (G') and loss (G") moduli of hydrogels at 40 wt% and 50 wt%, demonstrating elastic dominance (G'>G") and enhanced mechanical strength with increased solid content and crosslinking density.

Swelling and Water-Uptake Behavior

The equilibrium swelling behavior of the hydrogels exhibited a strong dependence on copolymer architecture and solid content. At 40 wt%, the random copolymer hydrogel (r402/DTPA) showed a significantly lower swelling ratio compared to the block copolymer hydrogels (b129/DTPA, b258/DTPA, and b402/DTPA), indicating a denser and more homogeneous crosslinked network. This reduced water uptake is consistent with the higher crosslink density and superior mechanical resilience observed in rheological measurements. In contrast, block copolymer hydrogels exhibited higher swelling ratios, which can be attributed

to microphase separation between HEMA-rich and NIPAM-rich domains and the selective participation of HEMA segments in DTPA-mediated crosslinking. The presence of relatively un-crosslinked NIPAM domains facilitates greater water penetration and network expansion. Increasing the solid content to 50 wt% resulted in a noticeable reduction in swelling for all formulations, reflecting the formation of denser polymer networks with restricted chain mobility. Overall, the swelling results correlate well with SEM observations and mechanical performance, further confirming that random copolymer architectures promote tighter network formation, while block copolymers yield more hydrated and heterogeneous gel structures.

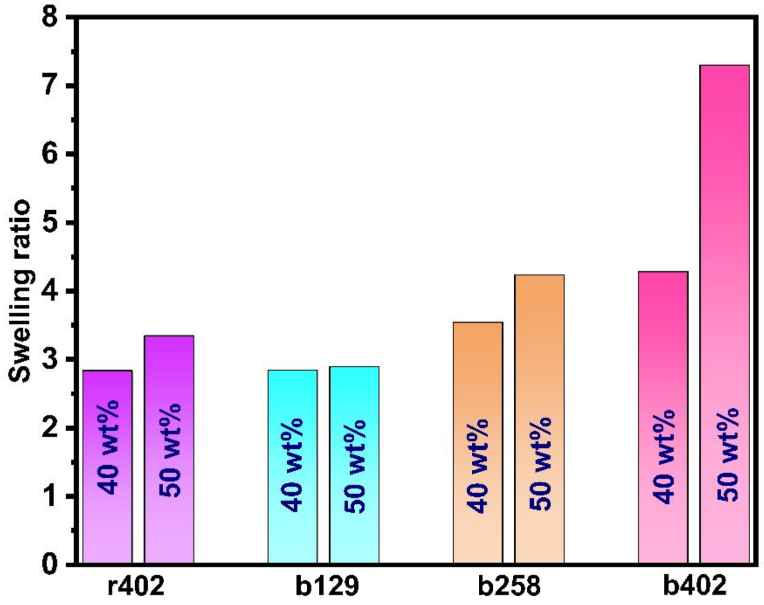

Fig. S3 Bar graph explanation for swelling ratio comparison between different hydrogels

Viscosity behavior

The solution viscosity of the copolymers exhibited a strong dependence on polymer architecture. At identical polymer loadings, random copolymers consistently showed higher viscosities than their block copolymer counterparts. This behavior is attributed to the uniform distribution of hydrophilic HEMA and thermo-responsive NIPAM units along the random copolymer backbone, which enhances intermolecular hydrogen bonding and increases effective chain entanglement in solution. In contrast, block copolymers displayed lower

viscosities at comparable concentrations, likely due to partial microphase segregation between HEMA-rich and NIPAM-rich segments, which reduces interchain interactions and limits entanglement density. These viscosity differences directly influence gelation kinetics and final network properties, as higher solution viscosity promotes more effective crosslinking and contributes to the superior mechanical performance observed in random copolymer hydrogels.

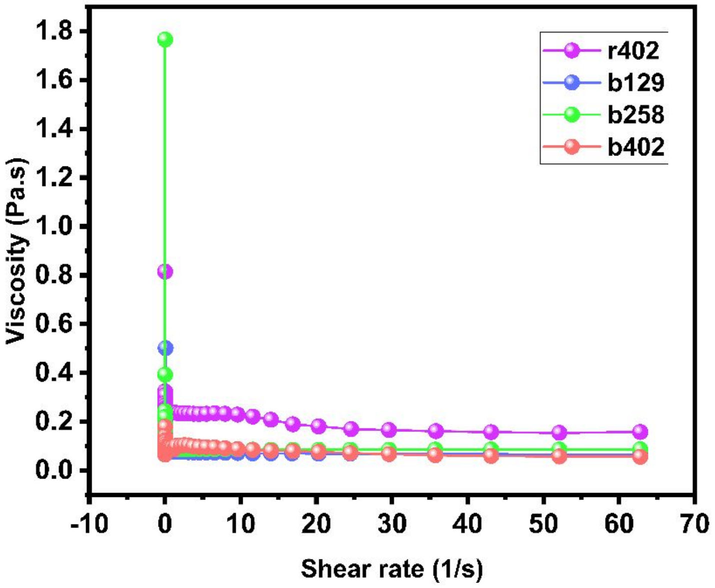

Fig. S4 Viscosity analysis of random and different block copolymers

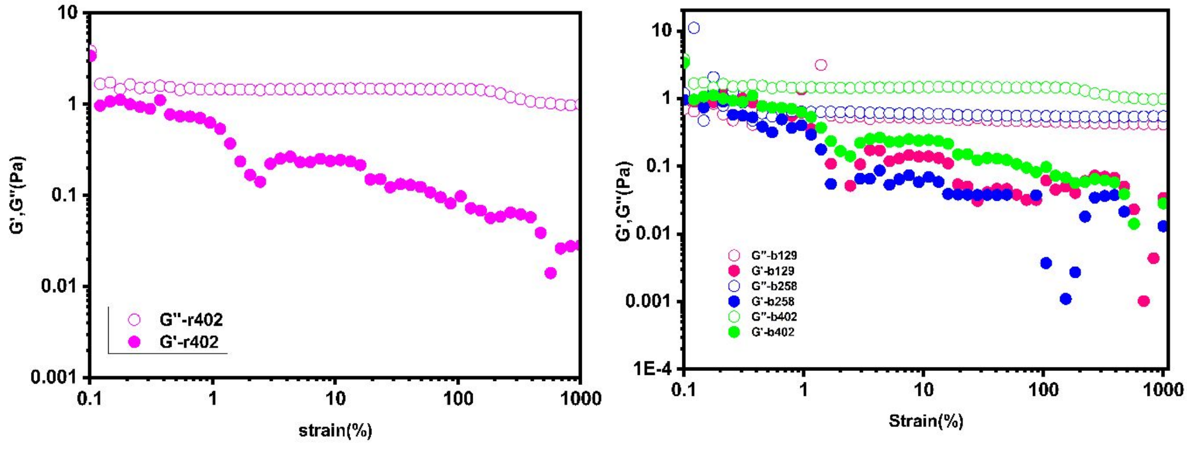

Fig. S5 Rheology analysis of random (r402) and different block copolymers without addition of any crosslinking agent
